# Quantifying the collective influence of social determinants of health using conditional and cluster modeling

**DOI:** 10.1371/journal.pone.0241868

**Published:** 2020-11-05

**Authors:** Zachary D. Rethorn, Alessandra N. Garcia, Chad E. Cook, Oren N. Gottfried

**Affiliations:** 1 Doctor of Physical Therapy Division, Duke University, Durham, North Carolina, United States of America; 2 Physical Therapy Program, College of Pharmacy & Health Sciences, Campbell University, Buies Creek, North Carolina, United States of America; 3 Duke Clinical Research Institute, Duke University, Durham, North Carolina, United States of America; 4 Department of Neurosurgery, Duke University Medical Center, Durham, North Carolina, United States of America; University of California San Francisco, UNITED STATES

## Abstract

**Objectives:**

Our objective was to analyze the collective effect of social determinants of health (SDoH) on lumbar spine surgery outcomes utilizing two different statistical methods of combining variables.

**Methods:**

This observational study analyzed data from the Quality Outcomes Database, a nationwide United States spine registry. Race/ethnicity, educational attainment, employment status, insurance payer, and gender were predictors of interest. We built two models to assess the collective influence of SDoH on outcomes following lumbar spine surgery—a stepwise model using each number of SDoH conditions present (0 of 5, 1 of 5, 2 of 5, etc) and a clustered subgroup model. Logistic regression analyses adjusted for age, multimorbidity, surgical indication, type of lumbar spine surgery, and surgical approach were performed to identify the odds of failing to demonstrate clinically meaningful improvements in disability, back pain, leg pain, quality of life, and patient satisfaction at 3- and 12-months following lumbar spine surgery.

**Results:**

Stepwise modeling outperformed individual SDoH when 4 of 5 SDoH were present. Cluster modeling revealed 4 distinct subgroups. Disparities between the younger, minority, lower socioeconomic status and the younger, white, higher socioeconomic status subgroups were substantially wider compared to individual SDoH.

**Discussion:**

Collective and cluster modeling of SDoH better predicted failure to demonstrate clinically meaningful improvements than individual SDoH in this cohort. Viewing social factors in aggregate rather than individually may offer more precise estimates of the impact of SDoH on outcomes.

## Introduction

Internationally, lumbar spine surgery is typically reserved for individuals who have responded poorly to conservative care or have marked physiological degeneration that has resulted in very high levels of pain, disability and lower levels of function [1, 2]. Because spine surgery also has higher incidences of harms and costs, a significant amount of effort has gone into modeling individuals who are good candidates for surgical intervention and conversely, those who are at risk for poor outcomes [3–5].

Scientists, clinicians and policy makers have recognized the influence of biopsychosocial factors on self-reported health outcomes[6]. Care pathways and risk stratification schemes commonly take into account biological and psychological factors [7, 8], yet noticeably less attention has been paid to social factors such as social determinants of health (SDoH). SDoH are broadly defined as the conditions in which people are born, work, live and play and include areas such as economic stability, education, social and community context, and environment [9]. Because recovery from spine surgery can be upwards of 6 months and correspond to increased psychological distress and decreased activity [10–13], we believe that the importance of addressing SDoH in this population is heightened.

To date, only small scale studies have evaluated individuals’ SDoH for spine surgery [4, 14–18], suggesting that these factors do individually influence outcomes. However, SDoH variables do not routinely exist in singularity. What remains unknown is the collective impact of SDoH on 3- and 12-month outcomes following lumbar spinal surgery. The goals of this study were to analyze the collective effect of SDoH on lumbar spine surgery outcomes utilizing two different statistical methods of combining variables. The findings will provide a better understanding of the role of SDoH, and will outline which method of statistical analysis defines a clearer picture of the role of the impact of SDoH on outcomes post lumbar surgery.

## Materials and methods

### Study design

This study was an observational design utilizing a retrospective review of a lumbar spine database from the Quality Outcomes Database (QOD). The QOD is a prospective registry established to define risk-adjusted morbidity and 12-month clinical outcomes following common surgical spine procedures [19, 20]. The registry has been enrolling patients since 2012 from 74 sites across 26 US states [20]. This study protocol was approved by the Duke University Institutional Review Board (Pro00029554) and adheres to the Reporting of studies Conducted using Observational Routinely collected Data (RECORD) guidelines [21].

### Participants

Patients aged 18 or older with degenerative disorders (stenosis, spondylolisthesis, disc herniation, scoliosis, kyphosis, or pseudarthrosis) who received a primary lumbar spine surgery (laminectomy, arthrodesis, osteotomy, corpectomy, interbody graft) were eligible for inclusion. Patients who received revision surgery and those who have reported baseline outcome values below minimum clinically important differences (MCIDs) for the study outcomes were excluded.

### Study variables

#### Descriptive variables

Patient characteristics at baseline included age, back pain, leg pain, disability, quality of life, gender, insurance payer, race, ethnicity, level of education, employment status, history of prior surgery, smoking status, and body mass index (BMI). Additional descriptive variables included baseline diagnoses of diabetes, coronary artery disease (CAD), peripheral vascular disease (PVD), anxiety, depression, chronic renal disease, or chronic obstructive pulmonary disease (COPD). Finally, baseline self-reported presence of pain, motor deficits, primary complaint, location of symptoms, duration of associated spine symptoms, American Society of Anesthesiologists (ASA) grade, and self-reported use of any pain medication were included.

#### Social determinant of health predictors

Five pre-operative SDoH variables were selected based on the Commission on Social Determinants of Health Final Report published by World Health Organization [22]. The variables included race/ethnicity, educational attainment, employment status, insurance payer, and gender, which were dichotomized based on previous research findings [4, 17, 23–26] to improve interpretability of findings.

The race responses of “American Indian or Alaska Native,” “Asian,” “Black or African American,” or “Native Hawaiian or Other Pacific Islander” were coded as responses of interest compared to “White.” The ethnicity response of “Hispanic or Latino” was coded as the response of interest compared to “non-Hispanic or Latino.” The race and ethnicity responses of interest were then aggregated together to create a combined variable of race and ethnicity.

The educational attainment response of “less than high school” was coded as the response of interest compared to “high school diploma or general equivalence diploma,” and any of the college experience choices. The employment responses of “employed but not working” or “unemployed” were coded as responses of interest compared to “employed and working.” The insurance payer response of “uninsured,” “Medicaid,” or “Medicare” among individuals who were <65 years old were coded as responses of interest compared to “Medicare,” “Veteran’s Affairs/Government,” or “private.” The gender response of “female” was coded as the response of interest compared to “male.”

#### Outcome variables

The outcome variables (dependent variables) included five variables: 1) back pain intensity, 2) leg pain intensity, 3) disability, 4) health status, and 5) patient satisfaction. Each variable was captured at 3 months and 12 months post-surgery. Back and leg pain intensity were measured using the 11-point numeric rating scale for back pain (NRS-BP) and the NRS for leg pain (NRS-LP) [27]. Pain intensity ratings range from 0 (no pain) to 10 (worst imaginable pain). Patients were asked to rate their pain on average of the last 7 days. Disability was measured using the Oswestry Disability Index (ODI) [28]. Quality of life was measured using the EuroQol five-dimensional questionnaire (EQ-5D) visual analog scale (VAS) [29]. This measure is a 0–100 scale with 0 representing the worst health imaginable and 100 representing the best health imaginable. Patients were asked to rate their health state on the evaluation day. These measures have been validated and are widely used in spine research [30–32]. Patient satisfaction was measured through the North American Spine Society patient satisfaction questionnaire [33], a four-point scale consisting of: “surgery met my expectations,” “I did not improve much but would undergo surgery for the same results,” “I did improve but would not undergo surgery for the same results,” and “I am the same or worse as compared to before surgery.”

Success thresholds were calculated from the change between baseline and each time point. Thresholds were defined for NRS-BP (1.2 points), NRS-LP (1.6 points), and ODI (12.8 points), using minimal clinically important difference (MCID) values previously reported [34]. To date, no MCID has been reported for the EQ-5D VAS. In this absence, we chose to use median change values (11 points for 3 months and 10 points for 12 months). Success in patient satisfaction was defined as either “Surgery met my expectations” or “I did not improve as much as I had hoped, but I would undergo the same operation for the same results.”

### Cohort derivation and missing data

Little’s missing completely at random (MCAR) test was employed for each variable and suggested that the data were not missing completely at random [35]. Methods for dealing with missing data can include listwise deletion and multiple imputation [36, 37]. Because the missing data were present in high-stakes variables, we chose to use listwise deletion to remove missing values in which an entire record is excluded from analysis if any single value is missing.

### Statistical analysis

Descriptive statistics were performed to assess differences in baseline variables utilizing linear mixed-effects modeling for continuous variables and Chi-square test for categorical variables [38].

#### Bivariate analyses

We ran independent analyses for each SDoH variable and each outcome variable. Age, multimorbidity (defined as 2 or more comorbid conditions) [39], surgical indication (spondylolisthesis, disc herniation, stenosis, scoliosis, kyphosis), type of surgery (laminectomy, arthrodesis, osteotomy, corpectomy, interbody graft), surgical approach (posterior only, anterior only, lateral only, two stage) and baseline outcome score were used as covariates as in similar studies [40–42]. The strength of association between the independent and dependent variables was expressed with adjusted odds ratios (ORs) with 95% confidence intervals (CIs) and Nagelkerke pseudo R-squared values that reflect the predictive power of the model [42, 43]. In our study, ORs above 1.0 indicated the likelihood of not meeting the MCID whereas ORs below 1.0 reflected the likelihood of meeting the MCID. The percentage of participants meeting each condition variable was calculated.

#### Statistical modeling method 1—Stepwise regression

To determine the associations between collective SDoH we utilized binary logistic regression between conditions of 0 of 5, 1 of 5, 2 of 5, 3 of 5, 4 of 5, and 5 of 5 SDoH and lumbar spine surgical outcomes adjusted for age, multimorbidity, surgical indication, type of surgery, surgical approach, and baseline outcome score as in the bivariate analyses. We converted the inverse of the odds ratios to probabilities of 100 patients achieving success and calculated the difference between individuals with 0 of 5 SDoH conditions and the 4 of 5 SDoH conditions.

#### Statistical modeling method 2—Cluster analysis

To better understand patterns of SDoH, we utilized a two-step cluster analysis to subgroup patients based upon the SDoH variables. Cluster analysis identifies homogenous subgroups who have similar characteristics where the grouping is not previously known [44]. The two-step cluster analysis first identifies groupings by pre-clustering based on dense regions in the attribute-space, then merges them using hierarchical methods [44]. We utilized the Bayesian Information Criterion (BIC) to determine the appropriate number of clusters that was based on the lowest BIC and the largest BIC change between the number of clusters [44]. Silhouette coefficients were used to appraise cluster solution quality with less than 0.2 classified as poor; between 0.2 and 0.5 as fair; greater than 0.5 as good solution quality. We considered good solution quality as acceptable clustering [44]. Two-step clustering has been regarded as a reliable and reproducible way to classify subgroups of individuals [45, 46].

We dummy coded each cluster and utilized binary logistic regression modeling to measure the associations between each clustered subgroup and lumbar spine surgical outcomes as in method 1. We converted the inverse of the odds ratios to probabilities of 100 patients achieving success and calculated the difference between subgroups. Significance was set at p < 0.05 and analyses were performed using R (R Foundation for Statistical Computing, Vienna, Austria; version 4.0.2) including the ‘rms’ package [47] and SPSS version 25.0 (IBM Corporation, Armonk, NY).

#### Sensitivity analyses

We performed sensitivity analyses (S1 Appendix) with missing values multiply-imputed using a flexible additive imputation model with predictive mean matching for missing values (n = 32,573). This method of imputation takes all aspects of uncertainty in the imputations into account by using the bootstrap to approximate the process of drawing predicted values from a full Bayesian predictive distribution [48]. Predictive mean matching works for binary, categorical, and continuous variables without the need for iterative maximum likelihood fitting for binary and categorical variables, and without the need for computing residuals or for curtailing imputed values to be in the range of actual data [48].

## Results

**Fig 1:**
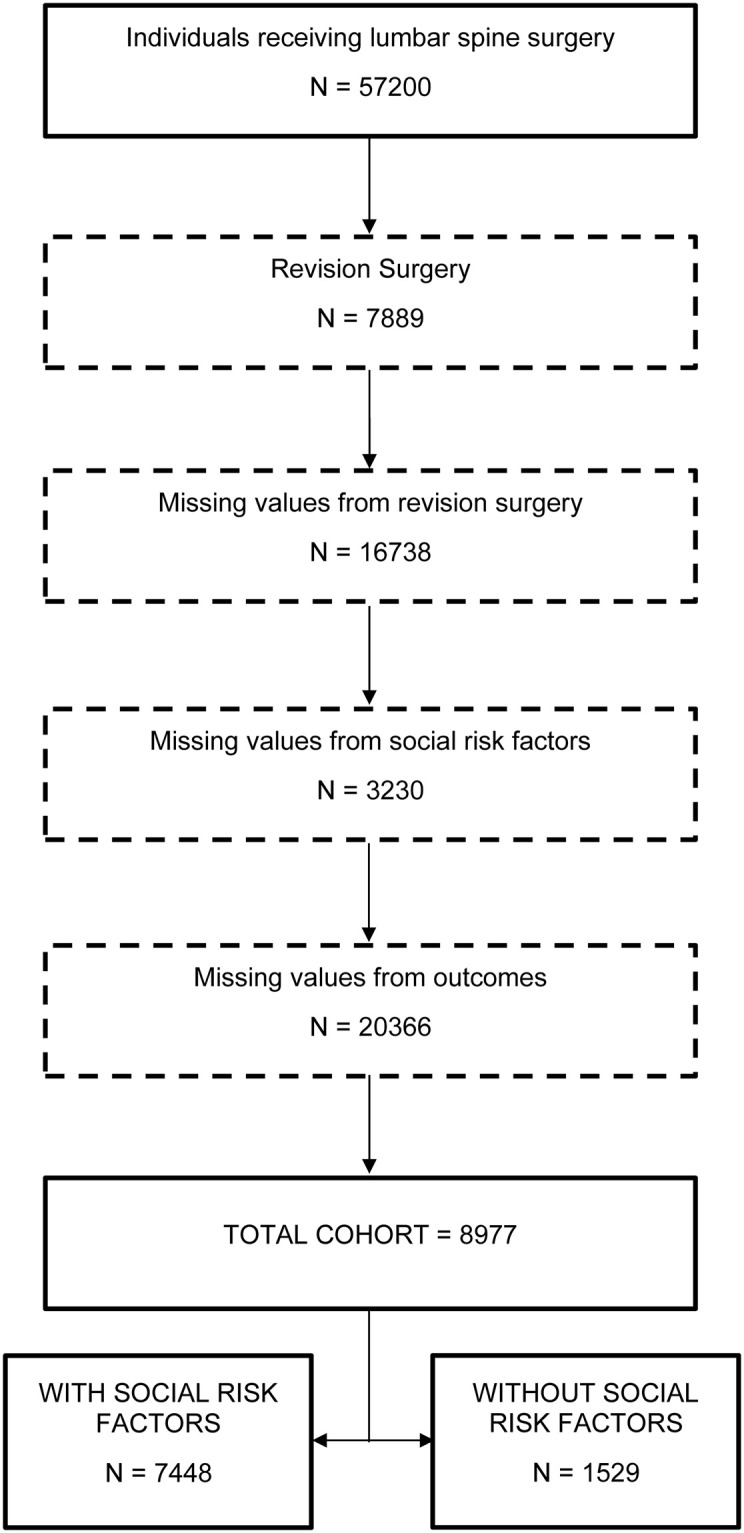
Cohort selection process.

Of the 8,977 individuals included in this study, 7,448 (83.0%) had SDoH whereas 1529 (17.0%) had none (Fig 1). Three thousand nine hundred and fifty-nine (44.1%) had two SDoH, 937 (10.4%) had three, 172 (1.9%) had four, and only 16 (0.2%) had five SDoH factors (S1 Table). Clustering identified four distinct subgroups: 1) older, white, female (OWF; n = 2249, 25.1%), 2) older, white, male (OWM; n = 2066, 23.0%) 3) younger, minority, low socioeconomic status (YML; n = 1952, 21.7%), and 4) younger, white, high socioeconomic status (YWH; n = 2710, 30.2%) with good cluster quality (average silhouette = 0.6). The overall trend was that the YML group had more pre-operative pain, disability, and comorbid conditions and lower QoL compared to the other groups. The YML cluster generally differed from the other groups in terms of level of education and insurance payer (Medicaid), but was similar in terms of baseline symptoms. However, those in the YML cluster were more likely to be smokers and have a higher BMI and COPD. Pre-operative characteristics of the four subgroups are described in Table 1.

**Table 1:** Baseline characteristics of study population and clustered subgroups.

Variable	Total Sample (n = 8977)	Older white female cluster (n = 2249)	Older white male cluster (n = 2066)	Younger minority lower cluster SES (n = 1952)	Younger white higher SES cluster (n = 2710)
**Sociodemographic**					
Mean age in years (SD)*	60.6 (13.62)	67.0 (11.3)	66.5 (12.0)	56.4 (12.9)	53.7 (12.6)
Gender*					
Male	4487 (50.0)	0 (0)	2066 (100)	892 (45.7)	1529 (56.4)
Female	4490 (50.0)	2249 (100)	0 (0)	1060 (54.3)	1181 (43.6)
White Race*	8117 (90.4)	2249 (100)	2066 (100)	1092 (55.9)	2710 (100)
Hispanic or Latino Ethnicity*	240 (2.7)	0 (0)	0 (0)	240 (12.3)	0 (0)
Level of education*					
Less than high school	412 (4.6)	0 (0)	0 (0)	412 (21.1)	0 (0)
High school diploma or GED	3823 (42.6)	1121 (49.8)	926 (44.8)	846 (43.3)	930 (34.3)
Two-year college degree	1675 (18.7)	422 (18.8)	365 (17.7)	332 (17.0)	556 (20.5)
Four-year college degree	1716 (19.1)	400 (17.8)	410 (19.8)	211 (10.8)	695 (25.6)
Post-college	1351 (15.0)	306 (13.6)	365 (17.7)	151 (7.7)	529 (19.5)
Insurance Payer*					
Uninsured	61 (0.70)	0 (0)	0 (0)	61 (3.1)	0 (0)
Medicare	3743 (41.7)	1390 (61.8)	1206 (58.4)	805 (41.2)	342 (12.6)
Medicaid	395 (4.4)	0 (0)	0 (0)	395 (20.2)	0 (0)
VA/Government	307 (3.4)	56 (2.5)	122 (5.9)	37 (1.9)	92 (3.4)
Private	4471 (49.8)	803 (35.7)	738 (35.7)	654 (33.5)	2276 (84.0)
Current employment status*					
Employed and currently working	3232 (36.0)	0 (0)	0 (0)	522 (26.7)	2710 (100)
Employed but not working	722 (8.0)	208 (9.2)	315 (15.2)	199 (10.2)	0 (0)
Unemployed	5023 (55.9)	2041 (90.8)	1751 (84.8)	1231 (63.1)	0 (0)
**Clinical/surgical**					
Mean baseline back pain (SD)*	7.15 (2.13)	7.29 (2.08)	7.00 (2.11)	7.74 (1.97)	6.71 (2.18)
Mean baseline leg pain (SD)*	7.32 (2.09)	7.43 (2.07)	7.08 (2.09)	7.72 (2.02)	7.13 (2.09)
Mean baseline disability (SD)*	48.9 (14.20)	50.1 (13.7)	46.9 (13.9)	53.9 (14.5)	45.8 (13.4)
Mean baseline quality of life (SD)*	59.98 (19.14)	59.6 (19.0)	60.1 (19.0)	57.6 (19.8)	62.0 (18.7)
Past surgery*	3476 (38.7)	958 (42.6)	918 (44.4)	705 (36.1)	895 (33.0)
Dominant symptom					
Pain	8628 (96.1)	2179 (96.9)	1969 (95.3)	1883 (96.5)	2597 (95.8)
Weakness	151 (1.7)	35 (1.6)	36 (1.7)	31 (1.6)	49 (1.8)
Numbness or tingling	176 (2.0)	32 (1.4)	56 (2.7)	32 (1.6)	56 (2.1)
Primary location of symptoms*					
Back	2007 (22.4)	484 (21.5)	431 (20.9)	493 (25.3)	599 (22.1)
Leg	2905 (32.4)	693 (30.8)	702 (34.0)	537 (27.5)	973 (35.9)
Back and Leg	4061 (45.2)	1072 (47.7)	932 (45.1)	920 (47.1)	1137 (42.0)
Symptom duration*					
> 3 months	7887 (87.9)	2013 (89.5)	1826 (88.4)	1743 (89.3)	2306 (85.1)
< 3 months	895 (10.0)	193 (8.6)	199 (9.6)	171 (8.8)	332 (12.3)
ASA grade*					
Grade 1	404 (4.5)	54 (2.4)	42 (2.0)	56 (2.9)	252 (9.3)
Grade 2	4552 (50.7)	1113 (49.5)	881 (42.6)	939 (48.1)	1619 (59.7)
Grade 3	3878 (43.2)	1047 (46.6)	1096 (53.0)	925 (47.4)	810 (29.9)
Grade 4	98 (1.1)	22 (1.0)	31 (1.5)	27 (1.4)	18 (0.7)
Grade 5	1 (0.0)	0 (0)	0 (0)	0 (0)	1 (0.0)
Surgical indication*					
Lumbar spondylolisthesis (grade 1)	2399 (26.7)	778 (34.6)	467 (22.6)	525 (26.9)	629 (23.2)
Lumbar disc herniation	3512 (39.1)	678 (30.1)	726 (35.1)	794 (40.7)	1314 (48.5)
Lumbar stenosis	5315 (59.2)	1376 (61.2)	1408 (68.2)	1129 (57.8)	1402 (51.7)
Posterior surgical approach	8255 (92.0)	2071 (92.1)	1915 (92.7)	1786 (91.5)	2483 (91.6)
Laminectomy/laminotomy performed	7822 (87.1)	1958 (87.1)	1830 (88.6)	1700 (87.1)	2334 (86.1)
Mean levels (SD)*	2.2 (0.9)	2.2 (0.9)	2.3 (0.9)	2.2 (0.9)	2.1 (0.8)
Arthrodesis/fusion performed*	3581 (39.9)	1137 (50.6)	708 (34.3)	786 (40.3)	950 (35.1)
Mean levels fused (SD)	1.5 (1.0)	1.6 (1.0)	1.6 (0.9)	1.6 (1.0)	1.5 (0.9)
Taking any pain medication*	7464 (83.1)	1888 (83.9)	1660 (80.3)	1668 (85.5)	2248 (83.0)
Discharge disposition*					
Home without services	7730 (86.1)	1756 (78.1)	1797 (87.0)	1630 (83.5)	2547 (94.0)
Home with services	574 (6.4)	193 (8.6)	127 (6.1)	160 (8.2)	94 (3.5)
Post-acute care	588 (6.6)	264 (11.7)	125 (6.1)	140 (7.2)	59 (2.2)
Acute care	79 (0.9)	35 (1.6)	16 (0.8)	20 (1.0)	8 (0.3)
**Comorbidities**					
Multimorbidity (≥2 comorbidities)*	7278 (81.1)	1829 (81.3)	1694 (82.0)	1640 (84.0)	2115 (78.0)
Smoker*	1297 (14.4)	208 (9.2)	291 (14.1)	447 (22.9)	351 (13.0)
BMI ≥ 30*	4533 (50.5)	1034 (46.0)	1026 (49.7)	1120 (57.4)	1353 (49.9)
Diabetes*	1825 (20.3)	470 (20.9)	509 (24.6)	507 (26.0)	339 (12.5)
CAD*	1079 (12.0)	225 (10.0)	436 (21.1)	211 (10.8)	207 (7.6)
PVD*	270 (3.0)	54 (2.4)	101 (4.9)	57 (2.9)	58 (2.1)
Anxiety*	1772 (19.7)	532 (23.7)	303 (14.7)	468 (24.0)	469 (17.3)
Depression*	2054 (22.9)	636 (28.3)	358 (17.3)	558 (28.6)	502 (18.5)
Arthritis*	2441 (27.2)	771 (34.3)	568 (27.5)	585 (30.0)	517 (19.1)
CKD*	307 (3.4)	87 (3.9)	103 (5.0)	71 (3.6)	46 (1.7)
COPD*	506 (5.6)	130 (5.8)	139 (6.7)	174 (8.9)	63 (2.3)
Osteoporosis*	470 (5.2)	257 (11.4)	38 (1.8)	100 (5.1)	75 (2.8)
Parkinson’s Disease*	50 (0.6)	15 (0.7)	22 (1.1)	5 (0.3)	8 (0.3)
Multiple Sclerosis	50 (0.6)	17 (0.8)	8 (0.4)	11 (0.6)	14 (0.5)
Pain (baseline)	8882 (98.9)	2226 (99.0)	2044 (98.9)	1926 (98.7)	2686 (99.1)
Motor deficits*	2749 (30.6)	663 (29.5)	660 (31.9)	632 (32.4)	794 (29.3)

*Significant difference between groups at *p* < 0.05.

Variables represent number (%) unless otherwise noted.

Abbreviations: SES, socioeconomic status; ODI, Oswestry Disability Index; EQ-5D, EuroQol-5D; VAS, visual analog scale; VA; Veteran’s Affairs; GED, General Equivalency Diploma; BMI, body mass index; CAD, coronary artery disease; PVD, peripheral vascular disease; CKD, chronic kidney disease; COPD, chronic obstructive pulmonary disease; ASA, American Society of Anesthesiologists.

### Individual SDoH

S2 Table presents the results of binary logistic regressions and outlines associations between each SDoH and failure to achieve success at 3 months. S3 Table presents similar results for 12-month outcomes. Statistically significant associations were noted between each SDoH and each outcome with the exception of gender. Overall, educational attainment, insurance type, and employment status were the strongest predictors of outcomes at 3 and 12 months. Sensitivity analyses revealed no substantial changes in 3-month outcomes (Table A in S1 Appendix) or 12-month outcomes (Table B in S1 Appendix).

### Stepwise modeling

S4 Table presents the conditional binary logistic regression results adjusted for age, multimorbidity, surgical indication, type of surgery, and baseline outcome score at 3 months. S5 Table displays similar results for 12-month outcomes. Statistically significant associations were noted for all outcomes at each time point. Overall, an additive effect for SDoH was observed across all outcome variables at 3- and 12-months post- surgery where the odds of failing to demonstrate success increased as more SDoH were present (S6 Table). The widest differences in outcomes were noted in patient satisfaction followed by leg pain and disability. S1 and S2 Figs show the difference in probability of 100 persons with 0 of 5 SDoH variables compared to those with 4 of 5 SDoH variables having success in each outcome at 3 and 12 months, respectively. Compared to those with 4/5 SDoH variables, between 19 and 31 more individuals with 0/5 SDoH variables out of 100 will experience success after lumbar spine surgery.

### Cluster modeling

**Fig 2:**
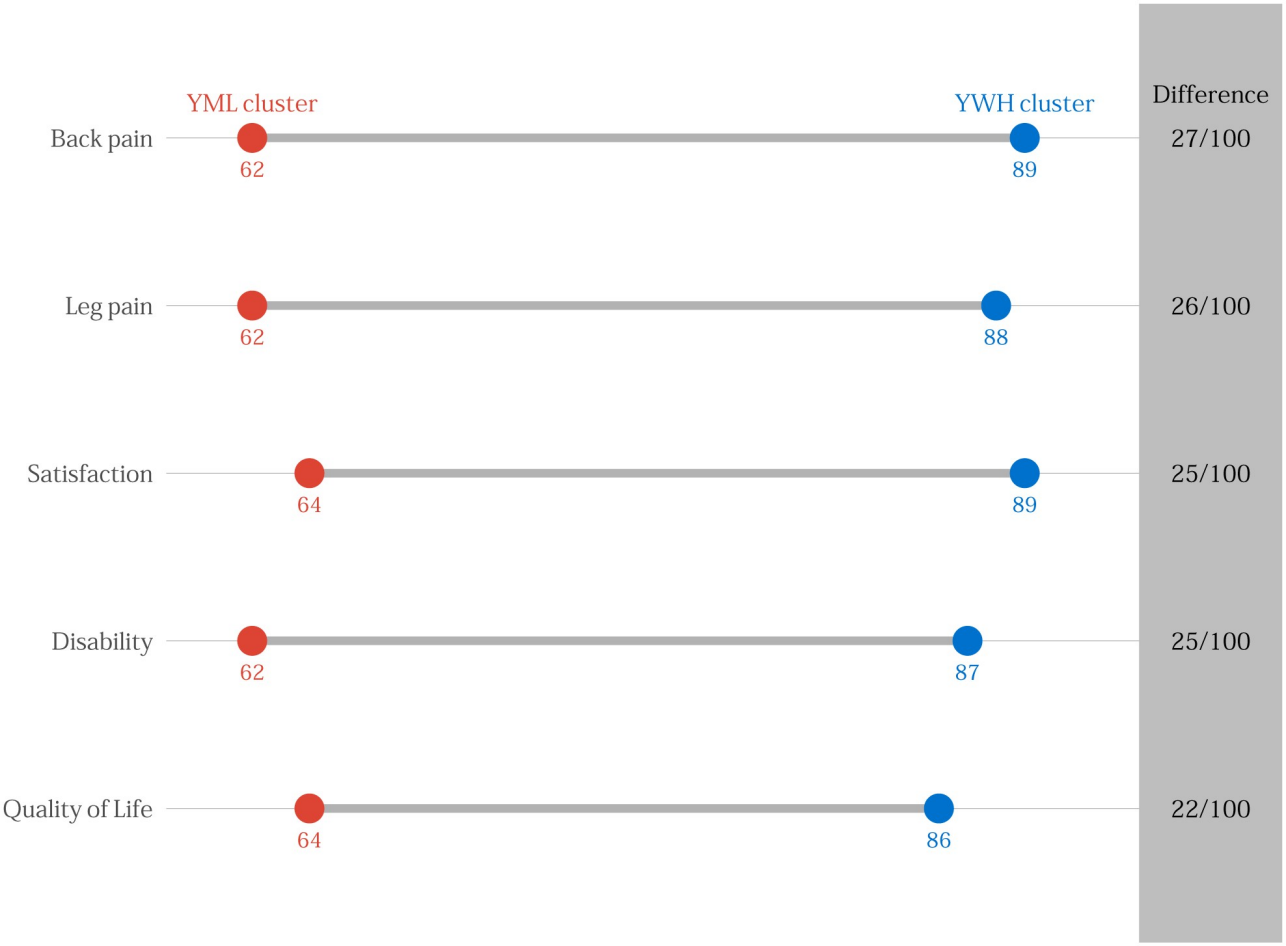
Probability of 100 persons from the younger, minority, low socioeconomic status (YML) subgroup compared to the younger, white, high socioeconomic status (YWH) subgroup achieving success on each outcome at 3 months.

**Fig 3:**
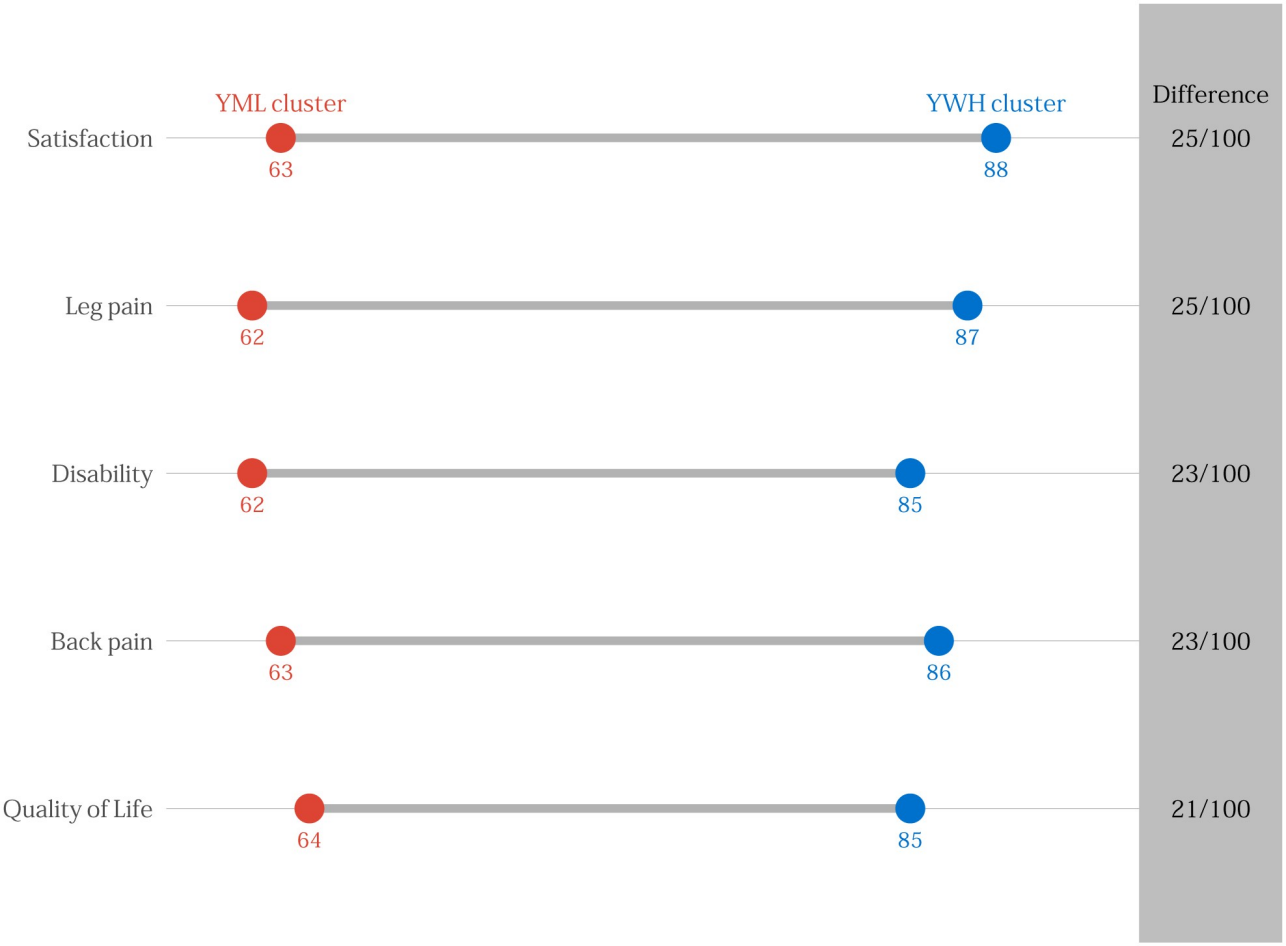
Probability of 100 persons from the younger, minority, low socioeconomic status (YML) subgroup compared to the younger, white, high socioeconomic status (YWH) subgroup achieving success on each outcome at 12 months.

Table 2 displays binary logistic regression results adjusted for age, multimorbidity, surgical indication, type of surgery, and baseline outcome score for each clustered subgroup. Table 3 presents similar findings for 12-month outcomes. S7 Table presents the mean baseline, 3-month, and 12-month outcomes by cluster. The OWF and OWM subgroups did not have statistically significant differences at 3 and 12 months. The YML subgroup demonstrated increased odds of failing to achieve success in each outcome at 3 and 12 months. In contrast, the YWH subgroup demonstrated decreased odds of failing to achieve success in each outcome at 3 and 12 months. The widest differences in outcomes were noted in back pain, leg pain, and disability. Figs 2 and 3 represent the differences between the probability of 100 persons in the YML compared to the YWH subgroup having success in each outcome at 3 and 12 months, respectively. Compared to individuals in the YML subgroup, between 21 and 27 more individuals from the YMH subgroup out of 100 will experience success after lumbar spine surgery.

**Table 2:** Association between clustered subgroup membership at baseline and failing to achieve clinically meaningful improvement on outcome at 3 months.

Outcome variable and clustered subgroup	Adjusted OR^†^ (95%CI)	*R*^*2*^	*p* value
MCID back pain (1.2 points, NRS, 0–10)			
OWF Cluster	1.06 (0.91, 1.23)	.125	.436
OWM Cluster	1.04 (0.91, 1.18)	.125	.900
YML Cluster	2.06 (1.78, 2.37)	.143	.**000**
YWH Cluster	0.49 (0.43, 0.57)	.143	.**000**
MCID leg pain (1.6 points, NRS, 0–10)			
OWF Cluster	1.09 (0.94, 1.27)	.080	.244
OWM Cluster	0.91 (0.79, 1.07)	.080	.276
YML Cluster	1.99 (1.72, 2.30)	.096	.**000**
YWH Cluster	0.51 (0.43, 0.59)	.095	.**000**
MCID disability (12.8 points, ODI, 0–100)			
OWF Cluster	0.96 (0.86, 1.08)	.120	.519
OWM Cluster	1.02 (0.92, 1.16)	.120	.623
YML Cluster	2.09 (1.85, 2.36)	.142	.**000**
YWH Cluster	0.53 (0.47, 0.59)	.139	.**000**
MCID quality of life (11 points, EQ-VAS, 0–100)			
OWF Cluster	1.05 (0.93, 1.20)	.377	.416
OWM Cluster	0.91 (0.80, 1.04)	.377	.149
YML Cluster	1.78 (1.57, 2.03)	.387	.**000**
YWH Cluster	0.54 (0.47, 0.62)	.387	.**005**
Patient satisfaction (2 points, 1–4)*			
OWF Cluster	0.99 (0.82, 1.19)	.010	.906
OWM Cluster	1.13 (0.95, 1.36)	.010	.176
YML Cluster	1.74 (1.47, 2.06)	.021	.**000**
YWH Cluster	0.49 (0.420, 0.60)	.025	.**000**

Abbreviations: MCID, minimal clinically important difference; CI, confidence interval; OR, odds ratio; OWF, older white female; OWM, older white male; YML, younger minority lower socioeconomic status; YWH, younger white higher socioeconomic status.

^†^Model was adjusted for age, the presence of multimorbidity, surgical indication, type of surgery, surgical approach, and baseline outcome score.

*Lower scores indicate higher satisfaction.

**Table 3:** Association between clustered subgroup membership at baseline and failing to achieve clinically meaningful improvement on outcome at 12 months.

Outcome variable and clustered subgroup	Adjusted OR^†^ (95%CI)	*R*^*2*^	*p* value
MCID back pain (1.2 points, NRS, 0–10)			
OWF Cluster	0.92 (0.80, 1.06)	.093	.246
OWM Cluster	1.12 (0.99, 1.28)	.093	.242
YML Cluster	1.93 (1.69, 2.20)	.110	.**000**
YWH Cluster	0.54 (0.48, 0.62)	.108	.**000**
MCID leg pain (1.6 points, NRS, 0–10)			
OWF Cluster	0.96 (0.83, 1.12)	.074	.608
OWM Cluster	0.94 (0.81, 1.09)	.074	.434
YML Cluster	2.09 (1.82, 2.40)	.095	.**000**
YWH Cluster	0.53 (0.46, 0.62)	.089	.**000**
MCID disability (12.8 points, ODI, 0–100)			
OWF Cluster	0.99 (0.89, 1.11)	.089	.924
OWM Cluster	0.92 (0.83, 1.04)	.089	.200
YML Cluster	2.03 (1.80, 2.29)	.111	.**000**
YWH Cluster	0.57 (0.51, 0.64)	.104	.**000**
MCID quality of life (10 points, EQ-VAS, 0–100)			
OWF Cluster	1.07 (0.94, 1.22)	.335	.310
OWM Cluster	0.88 (0.77, 1.01)	.335	.055
YML Cluster	1.70 (1.50, 1.92)	.343	.**000**
YWH Cluster	0.58 (0.51, 0.66)	.344	.**004**
Patient satisfaction (2 points, 1–4)*			
OWF Cluster	0.94 (0.81, 1.11)	.012	.478
OWM Cluster	1.07 (0.92, 1.25)	.012	.389
YML Cluster	1.90 (1.64, 2.20)	.029	.**000**
YWH Cluster	0.50 (0.42, 0.58)	.030	.**000**

Abbreviations: CI, confidence interval; OR, odds ratio; OWF, older white female; OWM, older white male; YML, younger minority lower socioeconomic status; YWH, younger white higher socioeconomic status.

^†^Model was adjusted for age, the presence of multimorbidity, surgical indication, type of surgery, surgical approach, and baseline outcome score.

*Lower scores indicate higher satisfaction.

## Discussion

This study analyzed the collective effect of SDoH on lumbar spine surgery outcomes by analyzing two different statistical methods of combining variables. We targeted individuals undergoing primary lumbar surgery in the hopes of homogenizing the patient population. When controlled for numerous covariates, across both types of modeling the presence of SDoH at baseline was associated with reduced success in improving in back pain, leg pain, disability, quality of life, and satisfaction at 3 and 12-month follow-up. These findings support the integration of SDoH for predictive modeling when determining prognosis following spine surgery. Interestingly, the findings associated with a collective effect when more than one SDoH variable was present was less definitive.

To our knowledge, this is the first study to examine 2 methods of modeling the collective impact of SDoH for any musculoskeletal disorder. Because social factors influence health in complex and interrelated ways [49], we elected to investigate two distinct methods of modeling the collective impact of SDoH for spine surgery. Whereas the stepwise regression modeling revealed an additive effect of SDoH (where each additional factor generally increased the odds of failing to demonstrate clinical improvement), it did not substantially outperform individual SDoH factors in predictive ability until 4 of 5 conditions were present. The clinical utility of this finding is limited since the number of patients with 4 of 5 of the measures SDoH represented only 1.9% of the overall sample.

**Fig 4:**
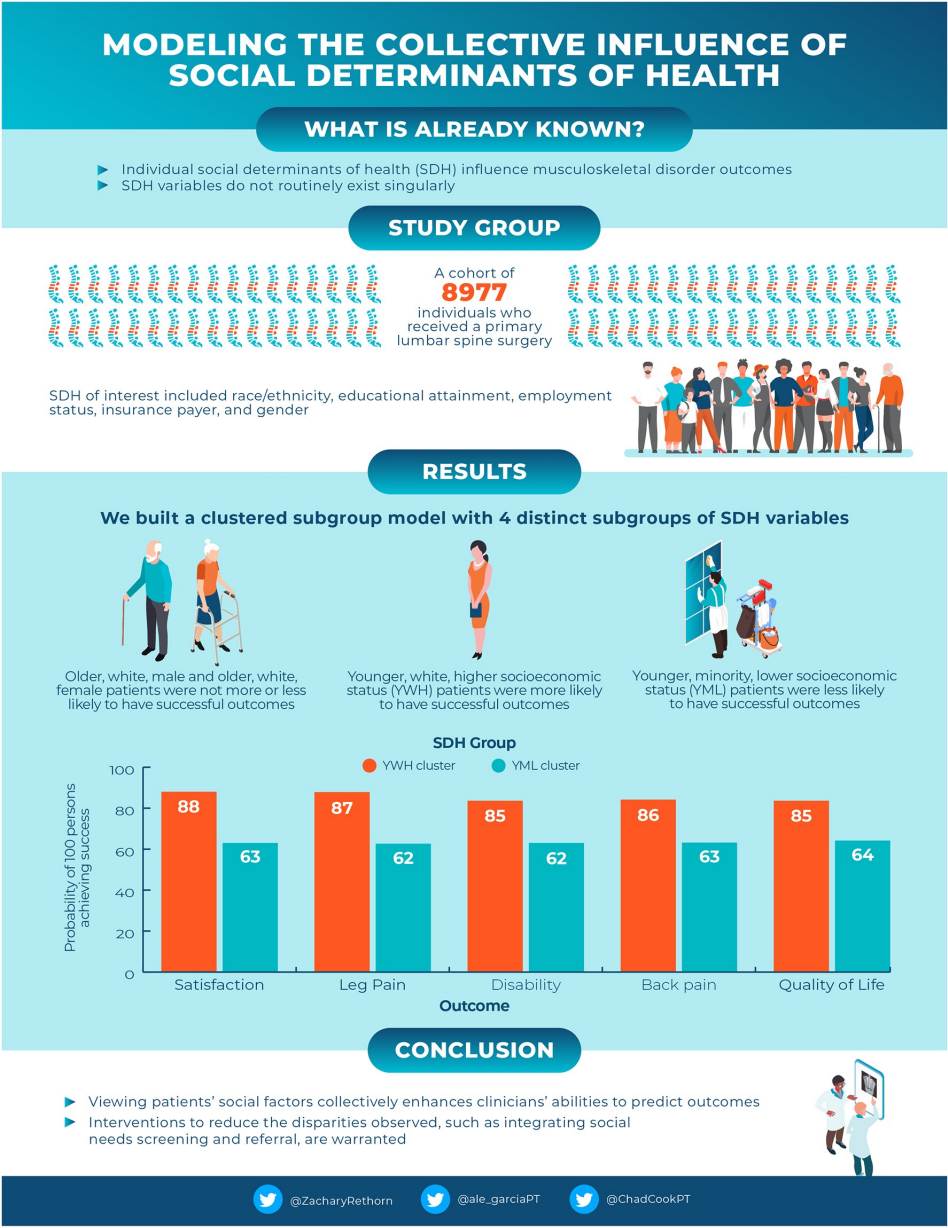
Infographic of quantifying the collective influence of social determinants of health.

The cluster modeling yielded intriguing results. The two-step cluster modeling identified four distinct patterns of SDoH: 1) OWF, 2) OWM, 3) YML, and 4) YWH. Sociodemographic, clinical, and comorbidity variables each differed by group allocation suggesting unique social-biological phenotypes. The differences observed between the YML and YWH subgroups were the most profound among the subgroups, especially with patient satisfaction, which exhibited the widest variation in success probability. The influence of various SDoH such as insurance and race on patient satisfaction has been previously documented in the surgical literature [17, 50]. The disparities seen in pain and disability have not previously been observed and begin to justify the need for more robust methods of quantifying the relationships between various SDoH [4, 51]. Overall, the cluster analysis produced subgroups with clearly defined characteristics that may be useful in clinical practice (Fig 4).

Lastly, the findings from this study shed light on potential care pathway structures for those who present with SDoH. Routine pre-operative screening for SDoH should be required to appropriately support patients.[52, 53] If a patient is a plausible candidate for surgery but has at least 3 of 5 SDoH variables implying risk or if the SDoH variables match the YML cluster identified, increased pre- and post-surgical community support may assist in mitigating the disparities observed in this study and optimize the risk benefit ratio in the patient’s favor. Prior studies have identified increased referral to and use of wraparound services including clinical team members or behavioral health when such pathways are implemented.[54, 55] Addressing SDoH in risk stratification models and care pathways is an important step toward improving the equity of outcomes from spine surgery.

### Limitations

This study is limited by its use of observational data in which cause and effect cannot be implied. Another limitation was the missing data present in the QOD. However, these missing data were handled through listwise deletion which is an acceptable procedure [56]. The definition of success was chosen based on standard MCID measures, but to date there are not universally agreed-on MCID values for all outcome measures [57]. The performance of the models based upon Nagelkerke pseudo R-squared value was modest to good with an explained variance of 1 to 38 percent. However, the utility of this measure in large behavior-based datasets has been called into question [58, 59]. Predictive models may be useful to guide clinician behavior even if the variance explained by the model is low. Finally, the 3- and 12-month time points are relatively short-term follow ups and the influence of SDoH at long-term time points remains unknown.

In this study, the predictor variable was developed by collapsing five variables—race/ethnicity, educational attainment, employment, insurance payer, and gender. Other social factors known to be associated with musculoskeletal disorders including income and place of residence were not available in the QOD [60, 61]. These data are especially important in light of recent work indicating that outcomes following microdiscectomy could not be accurately predicted by commonly captured sociodemographic variables [62]. It is unknown how including additional SDoH would affect the present results. Still, the authors hypothesize that the inclusion of additional SDoH variables would increase the precision and magnitude of the association between SDoH and clinical outcomes following spine surgery.

## Conclusion

The present study suggests that, in aggregate, SDoH predict failure to achieve success in pain, disability, quality of life, and satisfaction at 3- and 12-month follow-up time points following lumbar spinal surgery. Validation of these models in other populations with musculoskeletal disorders including robust markers of SDoH is warranted.

## Supporting information

S1 Fig Probability of 100 persons with 0 of 5 SDoH variables compared to those with 4 of 5 SDoH variables achieving success on each outcome at 3 months. (TIF)

S2 Fig Probability of 100 persons with 0 of 5 SDoH variables compared to those with 4 of 5 SDoH variables achieving success on each outcome at 12 months. (TIF)

S1 Table Baseline characteristics of study population by number of SDoH present. (DOCX)

S2 Table Association between presence of SDoH at baseline and failing to achieve clinically meaningful improvement on outcome at 3 months. (DOCX)

S3 Table Association between presence of SDoH at baseline and failing to achieve clinically meaningful improvement on outcome at 12 months. (DOCX)

S4 Table Association between presence of SDoH at baseline and failing to achieve clinically meaningful improvement on outcome at 3 months. (DOCX)

S5 Table Association between presence of SDoH at baseline and failing to achieve clinically meaningful improvement on outcome at 12 months. (DOCX)

S6 Table Baseline, 3-month, and 12-month outcomes for each SDoH condition. (DOCX)

S7 Table Baseline, 3-month, and 12-month outcomes for total sample and each cluster. (DOCX)

S1 Appendix Sensitivity analyses. (DOCX)
