# Supplementary figures and images for: Quantifying the collective influence of social determinants of health using conditional and cluster modeling
Source: PLoS One. 2020 Nov 5;15(11):e0241868. doi: 10.1371/journal.pone.0241868 (PMC7644039; doi:10.1371/journal.pone.0241868)

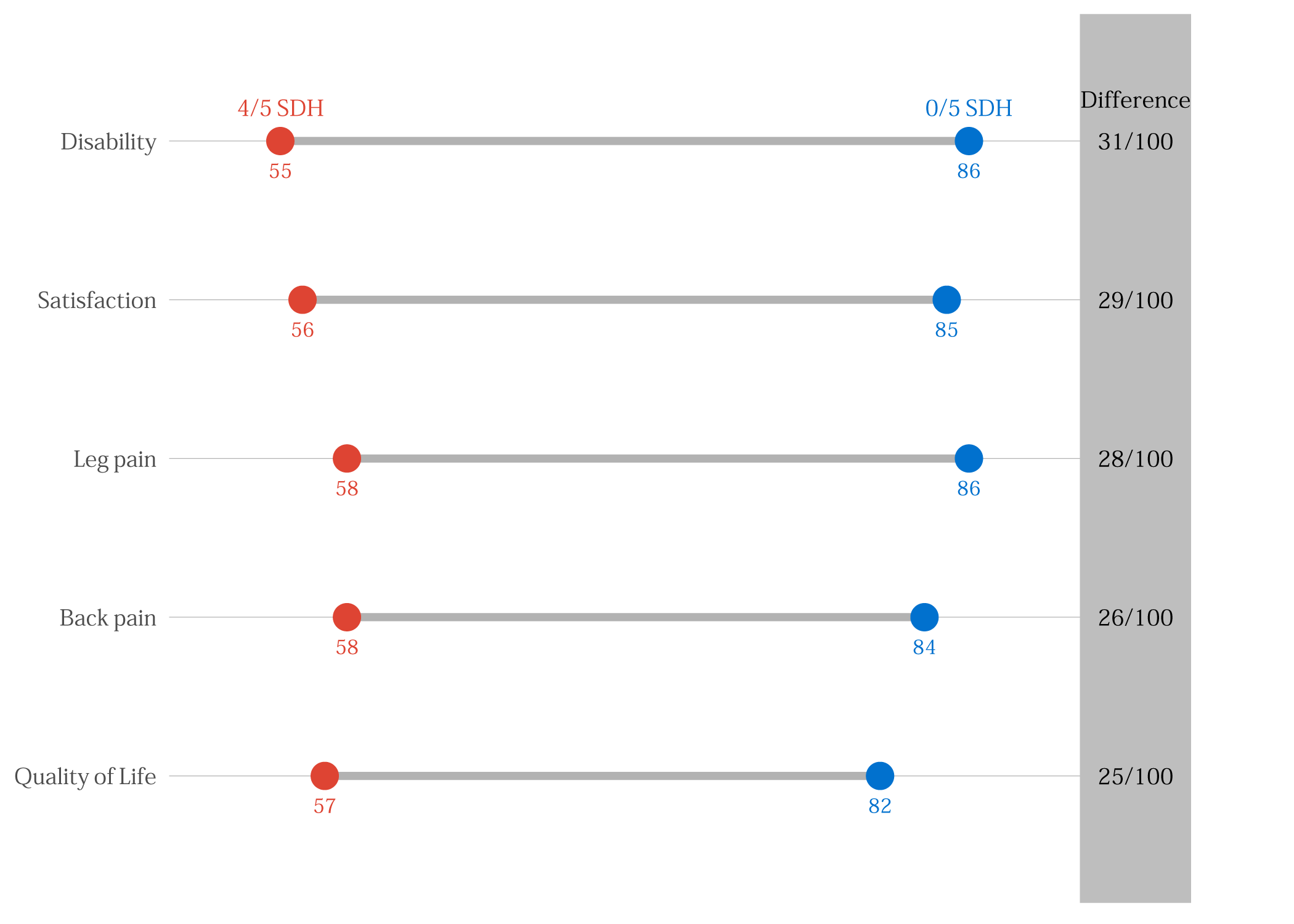

Supplement: S1 Fig — (TIF) [file pone.0241868.s001.tif]

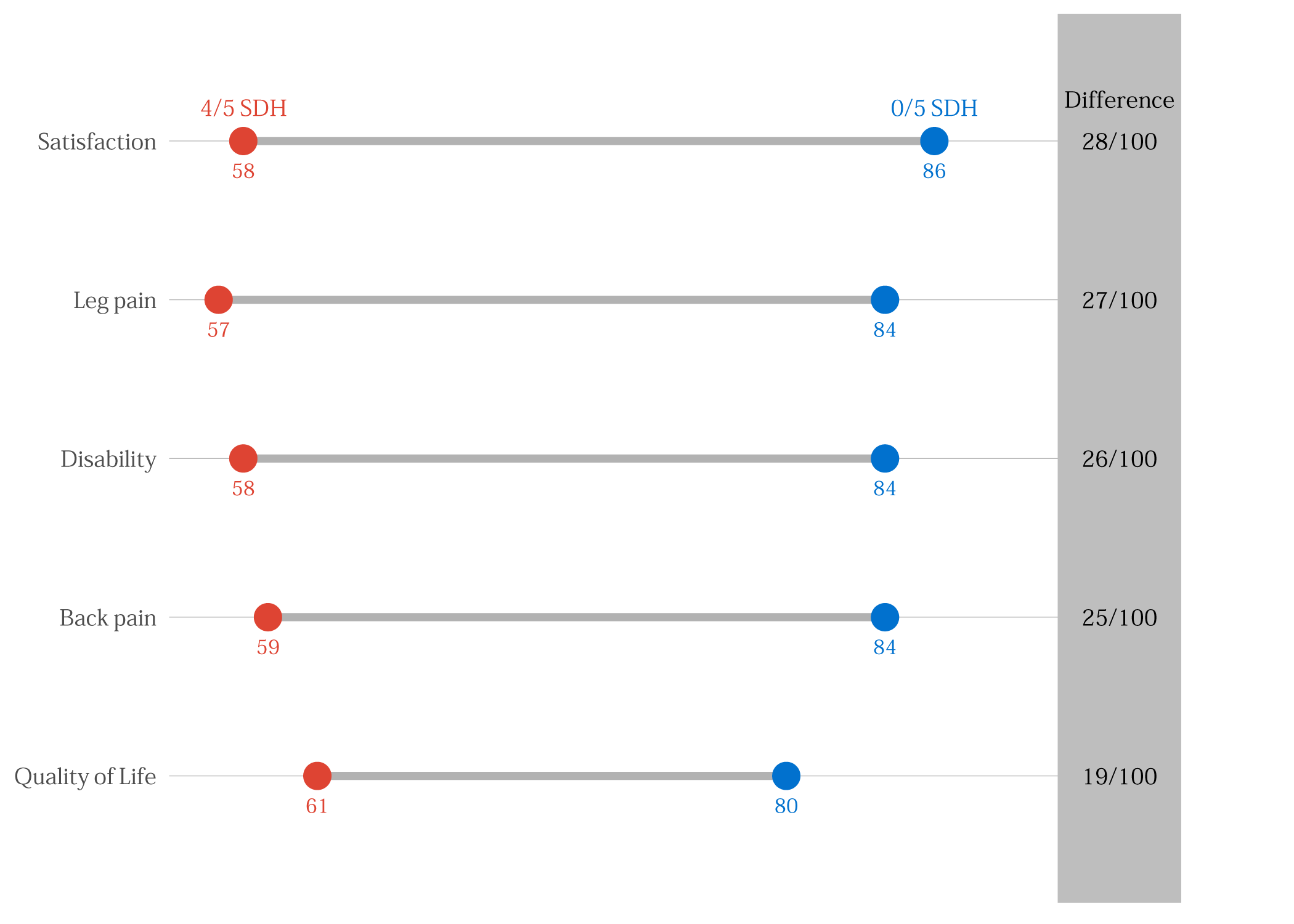

Supplement: S2 Fig — (TIF) [file pone.0241868.s002.tif]
