# Supplementary material for: Quantifying the collective influence of social determinants of health using conditional and cluster modeling
Source: PLoS One. 2020 Nov 5;15(11):e0241868. doi: 10.1371/journal.pone.0241868 (PMC7644039; doi:10.1371/journal.pone.0241868)
Supplement: S1 Table — (DOCX) [file pone.0241868.s003.docx]

**S1 Table. Baseline characteristics of study population by number of adverse SDH present**

| Variable | 0 of 5 SDH (n = 1529) | 1 of 5 SDH  (n = 7448) | 2 of 5 SDH  (n = 3959) | 3 of 5 SDH (n = 937) | 4 of 5 SDH  (n = 172) | 5 of 5 SDH (n = 16) |
| --- | --- | --- | --- | --- | --- | --- |
| **Sociodemographic** |  |  |  |  |  |  |
| Mean age in years (SD) | 60.6 (13.6) | 61.9 (13.4) | 62.8 (12.9) | 57.9 (12.5) | 55.2 (11.2) | 51.8 (10.5) |
| Gender |  |  |  |  |  |  |
| Male | 1529 (100) | 2958 (39.7) | 650 (16.4) | 115 (12.3) | 13 (7.6) | 0 (0) |
| Female | 0 (0) | 4490 (60.3) | 3309 (83.6) | 822 (87.7) | 159 (92.4) | 16 (100) |
| White Race | 1529 (100) | 6588 (88.5) | 3239 (81.8) | 538 (57.4) | 44 (25.6) | 0(0) |
| Hispanic or Latino Ethnicity | 0 (0) | 240 (3.2) | 189 (4.8) | 102 (10.9) | 20 (11.6) | 4 (25.0) |
| Level of education |  |  |  |  |  |  |
| Less than high school | 0 (0) | 412 (5.5) | 383 (9.7) | 255 (27.2) | 87 (50.6) | 16 (100) |
| High school diploma or GED | 549 (35.9) | 3274 (44.0) | 1866 (47.1) | 393 (41.9) | 57 (33.1) | 0(0) |
| Two-year college degree | 296 (19.4) | 1379 (18.5) | 715 (18.1) | 158 (16.9) | 14 (8.1) | 0(0) |
| Four-year college degree | 389 (25.4) | 1327 (17.8) | 574 (14.5) | 87 (9.3) | 9 (5.2) | 0(0) |
| Post-college | 295 (19.3) | 1056 (14.2) | 421 (10.6) | 44 (4.7) | 5 (2.9) | 0(0) |
| Insurance Payer |  |  |  |  |  |  |
| Uninsured | 0 (0) | 61 (0.8) | 49 (1.2) | 24 (2.6) | 3 (1.7) | 0(0) |
| Medicare | 208 (13.6) | 3535 (47.5) | 2171 (54.8) | 489 (52.2) | 85 (49.4) | 5 (31.2) |
| Medicaid | 0 (0) | 395 (5.3) | 369 (9.3) | 247 (26.4) | 79 (45.9) | 11 (68.8) |
| VA/Government | 66 (4.3) | 241 (3.2) | 83 (2.1) | 6 (0.6) | 0 (0) | 0(0) |
| Private | 1255 (82.1) | 3216 (43.2) | 1287 (32.5) | 171 (18.2) | 5 (2.9) | 0(0) |
| Current employment status |  |  |  |  |  |  |
| Employed and currently working | 1529 (100) | 1703 (22.9) | 280 (7.1) | 25 (2.7) | 1 (0.6) | 0 (0) |
| Employed but not working | 0 (0) | 722 (9.7) | 407 (10.3) | 105 (11.2) | 11 (6.4) | 1 (6.2) |
| Unemployed | 0 (0) | 5023 (67.4) | 3272 (82.6) | 807 (86.1) | 160 (93.0) | 15 (93.8) |
|  |  |  |  |  |  |  |
| **Clinical/surgical** |  |  |  |  |  |  |
| Mean baseline back pain (SD) | 6.53 (2.21) | 7.27 (2.09) | 7.53 (2.02) | 8.06 (1.80) | 8.36 (1.68) | 8.94 (1.18) |
| Mean baseline leg pain (SD) | 6.99 (2.10) | 7.39 (2.08) | 7.57 (2.05) | 7.92 (1.95) | 8.09 (1.88) | 8.56 (1.55) |
| Mean baseline disability (SD) | 44.6 (13.6) | 49.8 (14.2) | 52.2 (14.1) | 57.1 (14.0) | 59.9 (14.0) | 62.7 (13.2) |
| Mean baseline quality of life (SD) | 62.0 (18.3) | 59.6 (19.3) | 58.4 (19.4) | 55.2 (19.9) | 55.4 (18.6) | 44.3 (17.0) |
| Past surgery | 545 (35.6) | 2931 (39.4) | 1597 (40.3) | 341 (36.4) | 58 (33.7) | 4 (25.0) |
| Dominant symptom |  |  |  |  |  |  |
| Pain | 1463 (95.7) | 7187 (96.5) | 3841 (97.0) | 912 (97.3) | 168 (97.6) | 15 (93.8) |
| Weakness | 34 (2.2) | 117 (1.6) | 61 (1.5) | 12 (1.3) | 2 (1.2) | 0 (0) |
| Numbness or tingling | 32 (2.1) | 144 (1.9) | 57 (1.5) | 13 (1.4) | 2 (1.2) | 1 (6.2) |
| Primary location of symptoms |  |  |  |  |  |  |
| Back | 333 (21.8) | 1675 (22.5) | 919 (23.3) | 240 (25.6) | 51 (29.7) | 4 (25.0) |
| Leg | 569 (37.2) | 2337 (31.4) | 1138 (28.7) | 210 (22.4) | 30 (17.4) | 1 (6.2) |
| Back and Leg | 627 (50.0) | 3436 (46.1) | 1902 (48.0) | 487 (52.0) | 91 (52.9) | 11 (68.8) |
| Symptom duration |  |  |  |  |  |  |
| > 3 months | 1296 (84.8) | 6669 (89.5) | 3625 (91.6) | 874 (93.3) | 167 (97.1) | 14 (87.5) |
| < 3 months | 233 (15.2) | 779 (10.5) | 334 (8.4) | 63 (6.7) | 5 (2.9) | 2 (12.5) |
| ASA grade |  |  |  |  |  |  |
| Grade 1 | 159 (10.4) | 248 (3.3) | 84 (2.1) | 12 (1.3) | 2 (1.2) | 0 (0) |
| Grade 2 | 877 (57.3) | 3675 (49.4) | 1902 (48.1) | 403 (43.0) | 67 (39.0) | 5 (31.2) |
| Grade 3 | 480 (31.4) | 3439 (46.2) | 1925 (48.6) | 510 (54.4) | 100 (58.1) | 10 (62.5) |
| Grade 4 | 13 (0.9) | 85 (1.1) | 48 (1.2) | 12 (1.3) | 3 (1.7) | 1 (6.2) |
| Grade 5 | 0 (0) | 1 (0.0) | 0 (0) | 0 (0) | 0 (0) | 0 (0) |
| Surgical indication |  |  |  |  |  |  |
| Lumbar spondylolisthesis (grade 1) | 285 (18.6) | 2114 (28.4) | 1259 (31.8) | 307 (32.8) | 47 (27.3) | 2 (12.5) |
| Lumbar disc herniation | 759 (49.6) | 2753 (37.0) | 1326 (33.5) | 340 (36.3) | 77 (44.8) | 9 (56.2) |
| Lumbar stenosis | 831 (54.3) | 4484 (60.2) | 2374 (60.0) | 548 (58.5) | 91 (52.9) | 9 (56.2) |
| Posterior surgical approach | 1417 (92.7) | 6838 (91.8) | 3634 (91.8) | 862 (92.0) | 159 (92.4) | 16 (100) |
| Laminectomy/laminotomy performed | 1328 (86.9) | 6494 (87.2) | 3440 (86.9) | 817 (87.2) | 159 (92.4) | 15 (93.8) |
| Mean levels (SD) | 2.1 (0.9) | 2.2 (0.9) | 2.2 (0.9) | 2.1 (0.9) | 2.2 (1.0) | 2.2 (1.2) |
| Arthrodesis/fusion performed | 446 (29.2) | 3135 (42.1) | 1857 (46.9) | 419 (44.7) | 71 (41.3) | 6 (37.5) |
| Mean levels fused (SD) | 1.5 (0.8) | 1.6 (1.0) | 1.6 (1.0) | 1.6 (1.0) | 1.6 (1.0) | 2.2 (1.9) |
| Taking any pain medication | 1249 (81.7) | 6215 (83.4) | 3359 (84.8) | 825 (88.0) | 155 (90.1) | 13 (81.3) |
| Discharge disposition |  |  |  |  |  |  |
| Home without services | 1457 (95.3) | 6273 (84.2) | 3153 (79.6) | 731 (78.0) | 122 (70.9) | 0 (0) |
| Home with services | 38 (2.5) | 536 (7.2) | 349 (8.8) | 98 (10.5) | 23 (13.4) | 14 (87.5) |
| Post-acute care | 30 (2.0) | 558 (7.5) | 399 (10.1) | 95 (10.1) | 24 (14.0) | 2 (12.5) |
| Acute care | 2 (0.1) | 77 (1.0) | 55 (1.4) | 12 (1.3) | 3 (1.7) | 0 (0) |
|  |  |  |  |  |  |  |
| **Comorbidities** |  |  |  |  |  |  |
| Multimorbidity (≥2 comorbidities) | 1185 (77.5) | 6093 (81.8) | 3276 (82.7) | 799 (85.3) | 151 (87.8) | 14 (87.5) |
| Smoker | 217 (14.2) | 1080 (14.5) | 610 (15.4) | 224 (23.9) | 57 (33.1) | 6 (37.5) |
| BMI ≥ 30 | 775 (50.7) | 3758 (50.5) | 2032 (51.3) | 574 (61.3) | 113 (65.7) | 10 (62.5) |
| Diabetes | 207 (13.5) | 1618 (21.7) | 940 (23.7) | 279 (29.8) | 60 (34.9) | 6 (37.5) |
| CAD | 149 (9.7) | 930 (12.5) | 422 (10.7) | 92 (9.8) | 24 (14.0) | 3 (18.8) |
| PVD | 40 (2.6) | 230 (3.1) | 108 (2.7) | 26 (2.8) | 13 (7.6) | 2 (12.5) |
| Anxiety | 201 (13.1) | 1571 (21.1) | 975 (24.6) | 288 (30.7) | 46 (26.7) | 3 (18.8) |
| Depression | 194 (12.7) | 1860 (25.0) | 1169 (29.5) | 350 (37.4) | 62 (36.0) | 8 (50.0) |
| Arthritis | 275 (18.0) | 2166 (29.1) | 1318 (33.3) | 318 (33.9) | 67 (39.0) | 8 (50.0) |
| CKD | 28 (1.8) | 279 (3.7) | 156 (3.9) | 40 (4.3) | 14 (8.1) | 0 (0) |
| COPD | 42 (2.7) | 464 (6.2) | 300 (7.6) | 109 (11.6) | 23 (13.4) | 3 (18.8) |
| Osteoporosis | 11 (0.7) | 459 (6.2) | 356 (9.0) | 81 (8.6) | 13 (7.6) | 2 (12.5) |
| Parkinson’s Disease | 6 (0.4) | 44 (0.6) | 20 (0.5) | 0 (0) | 0 (0) | 0 (0) |
| Multiple Sclerosis | 4 (0.3) | 46 (0.6) | 28 (0.7) | 6 (0.6) | 3 (1.7) | 0 (0) |
| Pain (baseline) | 1515 (99.1) | 7367 (98.9) | 3913 (98.8) | 919 (98.1) | 169 (98.3) | 15 (93.8) |
| Motor deficits | 438 (28.6) | 2311 (31.0) | 1205 (30.4) | 311 (33.2) | 62 (36.0) | 4 (25.0) |

Variables represent number (%) unless otherwise noted.

Abbreviations: SES, socioeconomic status; ODI, Oswestry Disability Index; EQ-5D, EuroQol-5D; VAS, visual analog scale; VA; Veteran’s Affairs; GED, General Equivalency Diploma; BMI, body mass index; CAD, coronary artery disease; PVD, peripheral vascular disease; CKD, chronic kidney disease; COPD, chronic obstructive pulmonary disease; ASA, American Society of Anesthesiologists
