# Supplementary material for: Quantifying the collective influence of social determinants of health using conditional and cluster modeling
Source: PLoS One. 2020 Nov 5;15(11):e0241868. doi: 10.1371/journal.pone.0241868 (PMC7644039; doi:10.1371/journal.pone.0241868)
Supplement: S2 Table — (DOCX) [file pone.0241868.s004.docx]

**S2 Table. Association between presence of SDoH at baseline and failing to achieve clinically meaningful improvement on outcome at 3 months**

| Outcome variable and social determinant of health present | Adjusted OR^†^ (95%CI) | *R^2^* | *p* value |
| --- | --- | --- | --- |
| MCID back pain  (1.2 points, NRS, 0-10) |  |  |  |
| Race/ethnicity | 1.64 (1.37, 1.97) | .130 | **.000** |
| Education | 1.86 (1.43, 2.42) | .129 | **.000** |
| Insurance | 2.49 (2.06, 3.02) | .141 | **.000** |
| Employment | 2.10 (1.80, 2.44) | .142 | **.000** |
| Gender | 1.09 (0.97, 1.23) | .125 | .164 |
| MCID leg pain  (1.6 points, NRS, 0-10) |  |  |  |
| Race/ethnicity | 1.53 (1.27, 1.85) | .083 | **.000** |
| Education | 1.97 (1.52, 2.54) | .084 | **.000** |
| Insurance | 2.43 (2.01, 2.95) | .095 | **.000** |
| Employment | 2.04 (1.74, 2.38) | .095 | **.000** |
| Gender | 1.20 (1.06, 1.36) | .081 | **.005** |
| MCID disability (12.8 points, ODI, 0-100) |  |  |  |
| Race/ethnicity | 1.65 (1.41, 1.92) | .126 | **.000** |
| Education | 1.65 (1.32, 2.06) | .123 | **.000** |
| Insurance | 2.99 (2.52, 3.56) | .145 | **.000** |
| Employment | 2.27 (1.99, 2.59) | .143 | **.000** |
| Gender | 1.21 (1.10, 1.33) | .122 | **.000** |
| MCID quality of life (11 points, EQ-VAS, 0-100) |  |  |  |
| Race/ethnicity | 1.54 (1.30, 1.84) | .379 | **.000** |
| Education | 2.03 (1.57, 2.62) | .380 | **.000** |
| Insurance | 2.28 (1.88, 2.76) | .385 | **.000** |
| Employment | 2.20 (1.90, 2.55) | .390 | **.000** |
| Gender | 1.01 (0.90, 1.12) | .377 | .884 |
| Patient satisfaction  (2 points, 1-4)* |  |  |  |
| Race/ethnicity | 1.44 (1.16, 1.79) | .013 | **.001** |
| Education | 2.11 (1.60, 2.80) | .016 | **.000** |
| Insurance | 2.38 (1.92, 2.95) | .025 | **.000** |
| Employment | 1.69 (1.40, 2.03) | .018 | **.000** |
| Gender | 1.07 (0.92, 1.25) | .010 | .386 |

Abbreviations: MCID, minimal clinically important difference; CI, confidence interval; OR, odds ratio

^†^Model was adjusted for age, the presence of multimorbidity, surgical indication, type of surgery, surgical approach, and baseline outcome score

*Lower scores indicate higher satisfaction
