# Supplementary material for: Quantifying the collective influence of social determinants of health using conditional and cluster modeling
Source: PLoS One. 2020 Nov 5;15(11):e0241868. doi: 10.1371/journal.pone.0241868 (PMC7644039; doi:10.1371/journal.pone.0241868)
Supplement: S3 Table — (DOCX) [file pone.0241868.s005.docx]

**S3 Table. Association between presence of SDoH at baseline and failing to achieve clinically meaningful improvement on outcome at 12 months**

| Outcome variable and social determinant of health present | Adjusted OR^†^ (95%CI) | *R^2^* | *p* value |
| --- | --- | --- | --- |
| MCID back pain  (1.2 points, NRS, 0-10) |  |  |  |
| Race/ethnicity | 1.47 (1.24, 1.75) | .096 | **.000** |
| Education | 2.20 (1.74, 2.79) | .100 | **.000** |
| Insurance | 2.28 (1.91, 2.72) | .107 | **.000** |
| Employment | 1.98 (1.72, 2.27) | .109 | **.000** |
| Gender | 1.10 (0.99, 1.23) | .093 | .090 |
| MCID leg pain  (1.6 points, NRS, 0-10) |  |  |  |
| Race/ethnicity | 1.77 (1.48, 2.11) | .082 | **.000** |
| Education | 2.03 (1.59, 2.60) | .080 | **.000** |
| Insurance | 2.36 (1.96, 2.84) | .090 | **.000** |
| Employment | 2.11 (1.82, 2.45) | .093 | **.000** |
| Gender | 1.15 (1.02, 1.30) | .075 | **.020** |
| MCID disability (12.8 points, ODI, 0-100) |  |  |  |
| Race/ethnicity | 1.60 (1.37, 1.85) | .095 | **.000** |
| Education | 1.70 (1.36, 2.12) | .092 | **.000** |
| Insurance | 2.74 (2.31, 3.24) | .111 | **.000** |
| Employment | 2.12 (1.86, 2.41) | .110 | **.000** |
| Gender | 1.20 (1.09, 1.32) | .091 | **.000** |
| MCID quality of life (10 points, EQ-VAS, 0-100) |  |  |  |
| Race/ethnicity | 1.44 (1.22, 1.71) | .337 | **.000** |
| Education | 1.58 (1.23, 2.04) | .336 | **.000** |
| Insurance | 2.08 (1.72, 2.52) | .342 | **.000** |
| Employment | 2.13 (1.84, 2.47) | .348 | **.000** |
| Gender | 1.06 (0.96, 1.19) | .335 | .249 |
| Patient satisfaction  (2 points, 1-4)* |  |  |  |
| Race/ethnicity | 1.69 (1.41, 2.03) | .019 | **.000** |
| Education | 1.72 (1.33, 2.23) | .016 | **.000** |
| Insurance | 2.12 (1.75, 2.57) | .025 | **.000** |
| Employment | 1.93 (1.65, 2.26) | .027 | **.000** |
| Gender | 1.06 (0.93, 1.21) | .012 | .344 |

Abbreviations: MCID, minimal clinically important difference; CI, confidence interval; OR, odds ratio

^†^Model was adjusted for age, the presence of multimorbidity, surgical indication, type of surgery, surgical approach, and baseline outcome score

*Lower scores indicate higher satisfaction
