# Supplementary material for: Quantifying the collective influence of social determinants of health using conditional and cluster modeling
Source: PLoS One. 2020 Nov 5;15(11):e0241868. doi: 10.1371/journal.pone.0241868 (PMC7644039; doi:10.1371/journal.pone.0241868)
Supplement: S4 Table — (DOCX) [file pone.0241868.s006.docx]

**S4 Table. Association between presence of SDoH at baseline and failing to achieve clinically meaningful improvement on outcome at 3 months**

| Outcome variable and number of social determinants of health present (conditional modeling) | Adjusted OR^†^ (95%CI) | *R^2^* | *p* value |
| --- | --- | --- | --- |
| MCID back pain  (1.2 points, NRS, 0-10) |  |  |  |
| 0 of 5 present | 0.60 (0.51, 0.72) | .132 | **.000** |
| 1 of 5 present | 1.65 (1.40, 1.96) | .132 | **.000** |
| 2 of 5 present | 1.80 (1.58, 2.04) | .141 | **.000** |
| 3 of 5 present | 2.14 (1.78, 2.58) | .136 | **.000** |
| 4 of 5 present | 3.17 (2.18, 4.60) | .131 | **.000** |
| 5 of 5 present | 6.09 (2.06, 17.99) | .127 | **.001** |
| MCID leg pain  (1.6 points, NRS, 0-10) |  |  |  |
| 0 of 5 present | 0.56 (0.47, 0.68) | .088 | **.000** |
| 1 of 5 present | 1.77 (1.47, 2.14) | .088 | **.000** |
| 2 of 5 present | 1.77 (1.56, 2.02) | .095 | **.000** |
| 3 of 5 present | 1.92 (1.59, 2.32) | .088 | **.000** |
| 4 of 5 present | 3.17 (2.19, 4.58) | .086 | **.000** |
| 5 of 5 present | 4.08 (1.33, 12.54) | .080 | **.014** |
| MCID disability (12.8 points, ODI, 0-100) |  |  |  |
| 0 of 5 present | 0.54 (0.48, 0.62) | .133 | **.000** |
| 1 of 5 present | 1.84 (1.61, 2.10) | .133 | **.000** |
| 2 of 5 present | 1.64 (1.48, 1.81) | .134 | **.000** |
| 3 of 5 present | 2.59 (2.20, 3.05) | .140 | **.000** |
| 4 of 5 present | 4.77 (3.26, 6.97) | .131 | **.000** |
| 5 of 5 present | 3.41 (1.09, 10.69) | .120 | **.036** |
| MCID quality of life (11 points, EQ-VAS, 0-100) |  |  |  |
| 0 of 5 present | 0.67 (0.58, 0.77) | .380 | **.000** |
| 1 of 5 present | 1.49 (1.29, 1.73) | .380 | **.000** |
| 2 of 5 present | 1.46 (1.31, 1.64) | .382 | **.000** |
| 3 of 5 present | 2.07 (1.72, 2.48) | .384 | **.000** |
| 4 of 5 present | 3.47 (2.33, 5.17) | .381 | **.000** |
| 5 of 5 present | 4.67 (1.39, 15.64) | .377 | **.013** |
| Patient satisfaction  (2 points, 1-4)* |  |  |  |
| 0 of 5 present | 0.57 (0.45, 0.72) | .017 | **.000** |
| 1 of 5 present | 1.76 (1.39, 2.23) | .017 | **.000** |
| 2 of 5 present | 1.54 (1.32, 1.79) | .018 | **.000** |
| 3 of 5 present | 1.84 (1.49, 2.28) | .018 | **.000** |
| 4 of 5 present | 4.45 (3.10, 6.40) | .024 | **.000** |
| 5 of 5 present | 3.40 (1.06, 10.94) | .011 | **.040** |

Abbreviations: CI, confidence interval; OR, odds ratio

^†^Model was adjusted for age, the presence of multimorbidity, surgical indication, type of surgery, surgical approach, and baseline outcome score

*Lower scores indicate higher satisfaction
