# Supplementary material for: Quantifying the collective influence of social determinants of health using conditional and cluster modeling
Source: PLoS One. 2020 Nov 5;15(11):e0241868. doi: 10.1371/journal.pone.0241868 (PMC7644039; doi:10.1371/journal.pone.0241868)
Supplement: S5 Table — (DOCX) [file pone.0241868.s007.docx]

**S5 Table. Association between presence of SDoH at baseline and failing to achieve clinically meaningful improvement on outcome at 12 months**

| Outcome variable and number of social determinants of health present (conditional modeling) | Adjusted OR^†^ (95%CI) | *R^2^* | *p* value |
| --- | --- | --- | --- |
| MCID back pain  (1.2 points, NRS, 0-10) |  |  |  |
| 0 of 5 present | 0.59 (0.51, 0.69) | .101 | **.000** |
| 1 of 5 present | 1.68 (1.44, 1.97) | .101 | **.000** |
| 2 of 5 present | 1.54 (1.37, 1.72) | .103 | **.000** |
| 3 of 5 present | 2.12 (1.79, 2.52) | .105 | **.000** |
| 4 of 5 present | 2.81 (1.97, 4.00) | .098 | **.000** |
| 5 of 5 present | 3.85 (1.31, 11.32) | .094 | **.014** |
| MCID leg pain  (1.6 points, NRS, 0-10) |  |  |  |
| 0 of 5 present | 0.60 (0.50, 0.71) | .081 | **.000** |
| 1 of 5 present | 1.67 (1.40, 1.99) | .081 | **.000** |
| 2 of 5 present | 1.69 (1.50, 1.91) | .088 | **.000** |
| 3 of 5 present | 2.10 (1.76, 2.51) | .087 | **.000** |
| 4 of 5 present | 3.70 (2.60, 5.26) | .084 | **.000** |
| 5 of 5 present | 6.01 (2.04, 17.64) | .076 | **.001** |
| MCID disability (12.8 points, ODI, 0-100) |  |  |  |
| 0 of 5 present | 0.60 (0.53, 0.69) | .098 | **.000** |
| 1 of 5 present | 1.66 (1.46, 1.89) | .098 | **.000** |
| 2 of 5 present | 1.65 (1.50, 1.83) | .105 | **.000** |
| 3 of 5 present | 2.24 (1.91, 2.62) | .105 | **.000** |
| 4 of 5 present | 3.16 (2.22, 4.51) | .096 | **.000** |
| 5 of 5 present | 4.53 (1.38, 14.86) | .090 | **.013** |
| MCID quality of life (10 points, EQ-VAS, 0-100) |  |  |  |
| 0 of 5 present | 0.73 (0.63, 0.84) | .337 | **.000** |
| 1 of 5 present | 1.38 (1.19, 1.59) | .337 | **.000** |
| 2 of 5 present | 1.38 (1.24, 1.54) | .339 | **.000** |
| 3 of 5 present | 1.74 (1.46, 2.09) | .340 | **.000** |
| 4 of 5 present | 2.17 (1.48, 3.18) | .337 | **.000** |
| 5 of 5 present | 2.26 (0.66, 7.75) | .335 | .196 |
| Patient satisfaction  (2 points, 1-4)* |  |  |  |
| 0 of 5 present | 0.56 (0.46, 0.69) | .020 | **.000** |
| 1 of 5 present | 1.78 (1.46, 2.17) | .020 | **.000** |
| 2 of 5 present | 1.57 (1.37, 1.78) | .023 | **.000** |
| 3 of 5 present | 1.81 (1.50, 2.18) | .021 | **.000** |
| 4 of 5 present | 2.94 (2.05, 4.20) | .019 | **.000** |
| 5 of 5 present | 3.28 (1.09, 9.82) | .013 | **.034** |

Abbreviations: CI, confidence interval; OR, odds ratio

^†^Model was adjusted for age, the presence of multimorbidity, surgical indication, type of surgery, surgical approach, and baseline outcome score

*Lower scores indicate higher satisfaction
