# Supplementary material for: Quantifying the collective influence of social determinants of health using conditional and cluster modeling
Source: PLoS One. 2020 Nov 5;15(11):e0241868. doi: 10.1371/journal.pone.0241868 (PMC7644039; doi:10.1371/journal.pone.0241868)
Supplement: S6 Table — (DOCX) [file pone.0241868.s008.docx]

**S6 Table. Baseline, 3-month, and 12-month outcomes for each SDoH condition**

| Variable | 0 of 5 SDH (n = 1529) | 1 of 5 SDH  (n = 7448) | 2 of 5 SDH  (n = 3959) | 3 of 5 SDH (n = 937) | 4 of 5 SDH  (n = 172) | 5 of 5 SDH (n = 16) |
| --- | --- | --- | --- | --- | --- | --- |
| Mean back pain pre-surgery (SD) | 6.53 (2.21) | 7.27 (2.09) | 7.53 (2.02) | 8.06 (1.80) | 8.36 (1.68) | 8.94 (1.18) |
| Mean back pain at 3 months post-surgery (SD) | 2.37 (2.27) | 3.08 (2.69) | 3.42 (2.83) | 4.32 (2.99) | 5.12 (2.93) | 6.44 (2.76) |
| Mean back pain at 12 months post-surgery (SD) | 2.52 (2.52) | 3.30 (2.92) | 3.61 (3.04) | 4.64 (3.12) | 5.40 (2.95) | 6.38 (2.60) |
|  |  |  |  |  |  |  |
| Mean leg pain pre-surgery (SD) | 6.99 (2.10) | 7.39 (2.08) | 7.57 (2.05) | 7.92 (1.95) | 8.09 (1.88) | 8.56 (1.55) |
| Mean leg pain at 3 months post-surgery (SD) | 1.92 (2.48) | 2.47 (2.92) | 2.75 (3.09) | 3.53 (3.33) | 4.72 (3.24) | 4.50 (3.56) |
| Mean leg pain at 12 months post-surgery (SD) | 1.96 (2.63) | 2.64 (3.05) | 2.92 (3.19) | 3.77 (3.46) | 4.76 (3.49) | 5.81 (4.12) |
|  |  |  |  |  |  |  |
| Mean disability pre-surgery (SD) | 44.6 (13.6) | 49.8 (14.2) | 52.2 (14.1) | 57.1 (14.0) | 59.9 (14.0) | 62.7 (13.2) |
| Mean disability at 3 months post-surgery (SD) | 17.4 (15.8) | 25.5 (18.9) | 28.6 (19.4) | 35.6 (20.2) | 42.2 (20.2) | 38.5 (19.5) |
| Mean disability at 12 months post-surgery (SD) | 16.2 (16.7) | 22.7 (118) | 24.6 (161) | 34.3 (21.5) | 39.8 (20.4) | 41.5 (20.3) |
|  |  |  |  |  |  |  |
| Mean quality of life pre-surgery (SD) | 62.0 (18.3) | 59.6 (19.3) | 58.4 (19.4) | 55.2 (19.9) | 55.4 (18.6) | 44.3 (17.0) |
| Mean quality of life at 3 months post-surgery (SD) | 77.8 (14.7) | 73.4 (18.1) | 71.8 (18.7) | 66.7 (20.6) | 60.8 (23.0) | 57.4 (20.6) |
| Mean quality of life at 12 months post-surgery (SD) | 77.4 (16.1) | 72.9 (18.9) | 71.2 (19.5) | 66.6 (20.8) | 62.7 (21.2) | 48.9 (21.3) |
