# Supplementary material for: Quantifying the collective influence of social determinants of health using conditional and cluster modeling
Source: PLoS One. 2020 Nov 5;15(11):e0241868. doi: 10.1371/journal.pone.0241868 (PMC7644039; doi:10.1371/journal.pone.0241868)
Supplement: S7 Table — (DOCX) [file pone.0241868.s009.docx]

**S7 Table. Baseline, 3-month, and 12-month outcomes for total sample and each cluster**

| Variable | Total Sample (n = 8977) | Older white female cluster  (n = 2249) | Older white male cluster  (n = 2066) | Younger minority lower cluster SES (n = 1952) | Younger white higher SES cluster  (n = 2710) |
| --- | --- | --- | --- | --- | --- |
| Mean back pain pre-surgery (SD) | 7.15 (2.13) | 7.29 (2.08) | 7.00 (2.11) | 7.74 (1.97) | 6.71 (2.18) |
| Mean back pain at 3 months post-surgery (SD) | 2.96 (2.63) | 2.91 (2.61) | 2.73 (2.50) | 3.96 (2.94) | 2.47 (2.31) |
| Mean back pain at 12 months post-surgery (SD) | 3.16 (2.87) | 3.05 (2.84) | 3.05 (2.75) | 4.22 (3.13) | 2.59 (2.57) |
|  |  |  |  |  |  |
| Mean leg pain pre-surgery (SD) | 7.32 (2.09) | 7.43 (2.07) | 7.08 (2.09) | 7.72 (2.02) | 7.13 (2.09) |
| Mean leg pain at 3 months post-surgery (SD) | 2.38 (2.86) | 2.29 (2.84) | 2.27 (2.73) | 3.22 (3.26) | 1.93 (2.51) |
| Mean leg pain at 12 months post-surgery (SD) | 2.52 (3.00) | 2.42 (2.93) | 2.42 (2.90) | 3.48 (3.37) | 2.00 (2.66) |
|  |  |  |  |  |  |
| Mean disability pre-surgery (SD) | 48.9 (14.20) | 50.1 (13.7) | 46.9 (13.9) | 53.9 (14.5) | 45.8 (13.4) |
| Mean disability at 3 months post-surgery (SD) | 24.2 (18.6) | 24.9 (17.6) | 23.0 (17.9) | 32.0 (20.7) | 18.8 (16.3) |
| Mean disability at 12 months post-surgery (SD) | 21.6 (108) | 19.0 (212) | 21.8 (19.4) | 30.6 (22.0) | 17.1 (17.0) |
|  |  |  |  |  |  |
| Mean quality of life pre-surgery (SD) | 60.0 (19.14) | 59.6 (19.0) | 60.1 (19.0) | 57.6 (19.8) | 62.0 (18.7) |
| Mean quality of life at 3 months post-surgery (SD) | 74.2 (17.6) | 74.7 (17.0) | 73.7 (17.5) | 68.9 (20.0) | 77.8 (15.3) |
| Mean quality of life at 12 months post-surgery (SD) | 73.7 (18.6) | 73.8 (18.2) | 72.7 (18.7) | 68.6 (20.6) | 78.0 (16.1) |
