# Supplementary material for: Quantifying the collective influence of social determinants of health using conditional and cluster modeling
Source: PLoS One. 2020 Nov 5;15(11):e0241868. doi: 10.1371/journal.pone.0241868 (PMC7644039; doi:10.1371/journal.pone.0241868)
Supplement: S1 Appendix — (DOCX) [file pone.0241868.s010.docx]

**S1 Appendix. Sensitivity analyses**

We performed sensitivity analyses with missing values multiply-imputed using a flexible additive imputation model with predictive mean matching for missing values (n = 32,573). Table A shows the results of the 3-month outcomes using multiply-imputed data. Table B shows the results of the 12-month outcomes using multiply-imputed data. Overall, the bivariate sensitivity analyses revealed no major changes in findings – we observed similar odds ratios and R^2^ values.

**Table A. Association between presence of SDoH at baseline and failing to achieve clinically meaningful improvement on outcome at 3 months using multiply imputed data (n=32,573)**

| Outcome variable and social determinant of health present | Adjusted OR^†^ (95%CI) | *R^2^* | *p* value |
| --- | --- | --- | --- |
| MCID back pain  (1.2 points, NRS, 0-10) |  |  |  |
| Race/ethnicity | 1.55 (1.40, 1.71) | .126 | **.000** |
| Education | 1.92 (1.63, 2.25) | .125 | **.000** |
| Insurance | 2.40 (2.09, 2.77) | .131 | **.000** |
| Employment | 2.27 (2.08, 2.48) | .143 | **.000** |
| Gender | 1.11 (1.04, 1.20) | .122 | **.003** |
| MCID leg pain  (1.6 points, NRS, 0-10) |  |  |  |
| Race/ethnicity | 1.57 (1.42, 1.75) | .087 | **.000** |
| Education | 1.87 (1.58, 2.20) | .086 | **.000** |
| Insurance | 2.19 (1.89, 2.53) | .090 | **.000** |
| Employment | 2.05 (1.87, 2.25) | .099 | **.000** |
| Gender | 1.17 (1.08, 1.26) | .083 | **.000** |
| MCID disability (12.8 points, ODI, 0-100) |  |  |  |
| Race/ethnicity | 1.64 (1.50, 1.79) | .107 | **.000** |
| Education | 1.63 (1.40, 1.89) | .102 | **.000** |
| Insurance | 2.28 (1.99, 2.60) | .109 | **.000** |
| Employment | 2.31 (2.12, 2.51) | .124 | **.000** |
| Gender | 1.12 (1.05, 1.19) | .100 | **.001** |
| MCID quality of life (11 points, EQ-VAS, 0-100) |  |  |  |
| Race/ethnicity | 1.34 (1.21, 1.48) | .390 | **.000** |
| Education | 1.76 (1.49, 2.08) | .390 | **.000** |
| Insurance | 1.84 (1.58, 2.13) | .391 | **.000** |
| Employment | 2.26 (2.06, 2.48) | .403 | **.000** |
| Gender | 0.97 (0.91, 1.04) | .388 | .447 |
| Patient satisfaction  (2 points, 1-4)* |  |  |  |
| Race/ethnicity | 1.53 (1.36, 1.72) | .012 | **.000** |
| Education | 1.88 (1.57, 2.24) | .012 | **.000** |
| Insurance | 2.04 (1.74, 2.38) | .014 | **.000** |
| Employment | 1.83 (1.65, 2.03) | .019 | **.000** |
| Gender | 1.06 (0.97, 1.15) | .007 | .229 |

Abbreviations: MCID, minimal clinically important difference; CI, confidence interval; OR, odds ratio

^†^Model was adjusted for age, the presence of multimorbidity, surgical indication, type of surgery, surgical approach, and baseline outcome score

*Lower scores indicate higher satisfaction

**Table B. Sensitivity analyses of the association between presence of SDoH at baseline and failing to achieve clinically meaningful improvement on outcome at 12 months using multiply imputed data (n=32,573)**

| Outcome variable and social determinant of health present | Adjusted OR^†^ (95%CI) | *R^2^* | *p* value |
| --- | --- | --- | --- |
| MCID back pain  (1.2 points, NRS, 0-10) |  |  |  |
| Race/ethnicity | 1.53 (1.36, 1.73) | .093 | **.000** |
| Education | 2.16 (1.81, 2.60) | .095 | **.000** |
| Insurance | 2.30 (1.93, 2.73) | .097 | **.000** |
| Employment | 2.10 (1.89, 2.33) | .108 | **.000** |
| Gender | 1.09 (1.00, 1.19) | .088 | **.043** |
| MCID leg pain  (1.6 points, NRS, 0-10) |  |  |  |
| Race/ethnicity | 1.65 (1.45, 1.87) | .076 | **.000** |
| Education | 1.74 (1.43, 2.12) | .073 | **.000** |
| Insurance | 2.09 (1.74, 2.51) | .076 | **.000** |
| Employment | 2.19 (1.96, 2.45) | .091 | **.000** |
| Gender | 1.13 (1.03, 1.24) | .070 | **.011** |
| MCID disability (12.8 points, ODI, 0-100) |  |  |  |
| Race/ethnicity | 1.47 (1.31, 1.65) | .079 | **.000** |
| Education | 1.56 (1.30, 1.87) | .077 | **.000** |
| Insurance | 2.22 (1.86, 2.63) | .082 | **.000** |
| Employment | 2.40 (2.16, 2.68) | .101 | **.000** |
| Gender | 1.07 (0.99, 1.17) | .075 | .086 |
| MCID quality of life (10 points, EQ-VAS, 0-100) |  |  |  |
| Race/ethnicity | 1.36 (1.20, 1.54) | .344 | **.000** |
| Education | 1.49 (1.21, 1.82) | .344 | **.000** |
| Insurance | 1.97 (1.63, 2.39) | .346 | **.000** |
| Employment | 2.25 (2.00, 2.52) | .358 | **.000** |
| Gender | 0.94 (0.87, 1.03) | .343 | .175 |
| Patient satisfaction  (2 points, 1-4)* |  |  |  |
| Race/ethnicity | 1.58 (1.38, 1.80) | .014 | **.000** |
| Education | 1.80 (1.48, 2.19) | .013 | **.000** |
| Insurance | 2.16 (1.80, 2.60) | .017 | **.000** |
| Employment | 1.86 (1.65, 2.09) | .022 | **.000** |
| Gender | 1.06 (0.96, 1.17) | .009 | .260 |

Abbreviations: MCID, minimal clinically important difference; CI, confidence interval; OR, odds ratio

^†^Model was adjusted for age, the presence of multimorbidity, surgical indication, type of surgery, surgical approach, and baseline outcome score

*Lower scores indicate higher satisfaction
